# Editorial: Brain development and the attention spectrum: volume II

**DOI:** 10.3389/fnhum.2023.1221380

**Published:** 2023-06-19

**Authors:** Itai Berger, Alan Leviton, Yael Leitner

**Affiliations:** ^1^Department of Pediatric Neurology, Assuta-Ashdod University Hospital, Ashdod, Israel; ^2^Faculty of Health Sciences, Ben-Gurion University of the Negev, Beer-Sheva, Israel; ^3^The Paul Baerwald School of Social Work and Social Welfare, The Hebrew University, Jerusalem, Israel; ^4^Harvard Medical School, Boston Children's Hospital, Boston, MA, United States; ^5^The Pediatric ADHD Clinic, Tel Aviv University, Tel Aviv, Israel; ^6^Tel Aviv Sourasky Medical Center, Tel Aviv, Israel

**Keywords:** ADHD, diabetes mellitus, electric stimulation, kidney, autism spectrum disorder, epilepsy

The neuro-developmental deficits of Attention-Deficit Hyperactivity Disorder (ADHD) including decreased attention span, hyperactivity, distractibility, and impulsivity are frequently observed in children, adolescents and adults with a range of conditions (American Psychiatric Association, 2013). These symptoms are non-specific and can be overlooked even by experienced professionals, leading to inaccurate diagnoses and potential harm to treatment outcome. ADHD is among the disorders where diagnosis can be particularly challenging due to the variability in the manifestation of symptoms across individuals and populations, making it difficult to estimate the prevalence and neurobiological mechanisms of the disorder (Faraone et al., 2021). Among populations with dual diagnoses (comorbidities), such as those with intellectual disability, autism spectrum disorders, epilepsy, and other chronic illnesses, assessment of attention can be challenging, leading to potential underestimation or overestimation of the prevalence of ADHD (Fisher et al., 2022).

An alternative approach to understanding ADHD is through a developmental perspective, where attention characteristics are seen as expressions of developmental processes. This approach can provide insights into how risk and protective factors impact attention development, as well as associated co-morbidities (Berger et al., 2013). This alternative approach is the basis of the Research Topic that seeks to explore the latest findings about the biological, neural, psychosocial, and behavioral correlates of brain development and attention spectrum.

By highlighting the latest understanding of attention as a spectrum, we aim to shed light on the mechanisms underlying its development and the challenges in diagnosis and treatment over the entire age range. With this knowledge, we hope to equip a wide range of professionals with the necessary tools to better serve our patients with attention-related disorders.

In the ELGAN Study sample of children born at extremely low gestational age (*n* = 734), Cochran et al. report (*n* = 734), contrary to previous studies, that maternal smoking during pregnancy and pregnancy associated hypertension are did not predict ADHD at 10 and/or 15 years.

Several studies in this issue describe specific aspects of ADHD and ADHD “symptoms” in chronic pediatric conditions. Lancrei et al. report that their cohort with type-1 diabetes mellitus had rates of ADHD symptoms (20%), more than double the prevalence of ADHD in otherwise healthy pediatric populations, highlighting that the negative influence on neuropsychiatric functioning likely begins sooner in the disease course than previously anticipated. The authors propose these findings are of large practical importance as ADHD symptoms are likely to impact routine everyday decisions crucial for diabetes management (Lancrei et al.).

Duquette et al. describe ADHD in children with Chronic Kidney Disease (CKD). Although the sample size was small, results from the current study provide some of the most comprehensive findings to date on the differential attention functioning of children with CKD. Taken together, these findings suggest the existence of specific types of attention dysfunction, particularly in the focus/execute, sustain, and encoding, components of attention (Duquette et al.).

Uliel-Sibony et al. provide a comprehensive literature review of the common co-occurrence of ADHD and epilepsy, and describe the latest research regarding possible etiologies explaining the common co-occurrence. This review looks at the influence of medications on both disorders and the recommended treatment approaches in accordance with the latest literature (Uliel-Sibony et al.).

Sabag and Geva provide a neurobiological perspective on the very significant comorbidity of attention in autism spectrum disorder (ASD), describing the framework of attention network activation in ASD, and suggesting that this neurobiological perspective be considered for further investigation in naturalistic settings.

Slobodin and Davidovitch highlight the associations and differences between children's self-reports, parents' and teachers' reports, and standardized continuous performance test (CPT) data, and discuss the clinical and ethical imperative of taking children's perspectives into account during ADHD diagnosis and treatment.

Novel treatment approach of trans-cranial electric stimulation combined with cognitive training is described by Dakwar-Kawar et al. providing initial support for the efficacy of this combined method in improving processing speed in the presence of mental fatigue in pediatric ADHD.

Dutta et al. complete the series with a bottom-up holistic approach to the psychopharmacology of ADHD in the context of recent models of attention and suggest that future studies are greatly needed to better appreciate the interactions among an ADHD diagnosis, stimulant treatment across the lifespan, and structure-function alterations in the aging brain.

We hope this Research Topic will update our readers on both research and clinical aspects of ADHD.

## Author contributions

IB, AL, and YL conceptualized and conducted the project, collected the study data, drafted the manuscript, and reviewed the final manuscript. All authors have read and approved the final manuscript.
